# Molecular Mechanism of Dihydroquercetin in Ameliorating Metabolic Dysfunction–Associated Steatotic Liver Disease: Insights Into the HIF‐1α/VEGF Pathway

**DOI:** 10.1002/fsn3.71529

**Published:** 2026-02-11

**Authors:** Xinyue Zhang, Han Zhang, Bing Wu, Pengfei Wu, Ao Shen, Cheng Zhang, Yuqiao Zeng, Yanjun Wang, Hao Xu, Yiyu He, Likun Wang

**Affiliations:** ^1^ School of Clinical Medicine Shandong Second Medical University Weifang China; ^2^ Infection Control Center Linyi People's Hospital Linyi China; ^3^ Jilin Jianwei Natural Biotechnology Co. Ltd. Changchun China; ^4^ Department of Cardiovascular Disease Renmin Hospital of Wuhan University Wuhan China

**Keywords:** dihydroquercetin, HIF‐1 signaling pathway, inflammatory response, metabolic dysfunction–associated steatotic liver disease, network pharmacology

## Abstract

Dihydroquercetin (DHQ) is a plant‐derived flavonoid with well‐documented antioxidant and anti‐inflammatory properties. This study aimed to elucidate the therapeutic mechanisms of DHQ in metabolic dysfunction‐associated steatotic liver disease (MASLD) by integrating network pharmacology with in vivo and in vitro experiments. Network pharmacology analysis identified potential DHQ targets associated with MASLD, followed by GO and KEGG enrichment analyses. A high‐fat diet‐induced murine MASLD model and a free fatty acid‐induced HepG2 cell steatosis model were employed to evaluate the effects of DHQ. Biochemical assays, ELISA, Oil Red O staining, RT‐qPCR, and Western blotting were used to assess lipid metabolism, inflammatory responses, and HIF‐1 signaling. Thirty‐seven overlapping targets between DHQ and MASLD were identified, with protein–protein interaction analysis highlighting key hub proteins and enrichment analyses implicating the HIF‐1 signaling pathway. In vivo, DHQ significantly reduced body and liver weight, improved serum lipid profiles and liver enzyme levels, and alleviated hepatic histopathological damage. Mechanistically, DHQ activated the LKB1–AMPK axis, inhibited ACC, reduced IL‐1β and TNF‐α production, and attenuated aberrant HIF‐1α/VEGF signaling. Consistently, DHQ decreased lipid accumulation and inflammatory cytokine expression in steatotic HepG2 cells, while CoCl_2_‐induced HIF‐1α stabilization partially reversed these protective effects. Collectively, these findings suggest that DHQ ameliorates lipid metabolic disorders and inflammation in MASLD by modulating the HIF‐1α/VEGF pathway, supporting its potential as a therapeutic candidate.

## Graphical abstract

Dihydroquercetin (DHQ) alleviates lipid metabolic dysfunction and hepatic inflammation in MASLD. Mechanistically, DHQ modulates the HIF‐1α/VEGF signaling pathway, contributing to improved liver homeostasis.

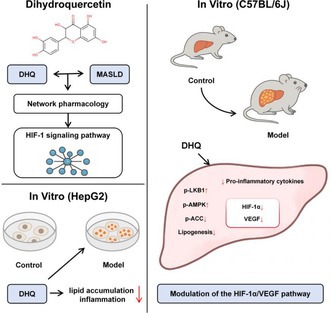

## 1 Introduction

Metabolic dysfunction‐associated steatotic liver disease (MASLD), characterized by excessive triglyceride accumulation in hepatocytes, is a rapidly growing metabolic disorder affecting about 25% of the global population (Huby and Gautier 2022; Lai et al. 2022; Martín‐Fernández et al. 2022). Despite its increasing prevalence and health impact, effective treatments remain lacking due to MASLD's complex pathogenesis involving metabolic dysregulation, inflammation, and oxidative stress (Barchetta et al. 2019). Natural compounds with antioxidant and anti‐inflammatory effects have gained attention for MASLD therapy, among which dihydroquercetin (DHQ), a flavonoid with hepatoprotective properties, shows promising preclinical efficacy (Abou‐Taleb et al. 2024; Wei et al. 2024).

Flavonoids are a diverse class of compounds abundant in traditional Chinese medicinal herbs. DHQ, a natural flavonoid derivative, is mainly extracted from plants such as *Allium cepa* , *Silybum marianum* , and *Pseudotsuga menziesii* (Huong et al. 2025; Weidmann 2012), and is widely used as a food additive in the health industry (Kalinina et al. 2023; Pan et al. 2024). DHQ exhibits multiple pharmacological effects including antiviral, anti‐inflammatory, antioxidant, and anti‐fibrotic activities (Rogovskii et al. 2021; Yuan et al. 2022; Trofimova et al. 2015; Liu et al. 2023). It can inhibit lipogenesis via the P2X7R‐inflammasome pathway and regulate lipid metabolism through AMPK‐dependent mechanisms, thereby reducing hepatic steatosis (Zhang et al. 2018). Additionally, DHQ shows anti‐fibrotic properties that protect liver function (Wei et al. 2024; Inoue et al. 2023). However, most existing studies primarily focus on DHQ‐mediated regulation of metabolic enzymes or inflammatory signaling pathways, while the upstream hypoxia‐related regulatory networks and angiogenic signaling involved in MASLD progression remain insufficiently investigated (Kounatidis et al. 2024). Therefore, whether DHQ exerts hepatoprotective effects by modulating hypoxia‐responsive pathways represents an important question.

Network pharmacology, which maps drug‐component‐target‐pathway interactions, offers insight into drug mechanisms and targets (Zheng et al. 2024; Ren et al. 2023). Hypoxia‐inducible factor‐1 (HIF‐1), a heterodimer of HIF‐1α and HIF‐1β, regulates genes involved in angiogenesis, glucose and lipid metabolism, and inflammation, playing a key role in cellular hypoxia adaptation (Seo et al. 2022; Shang et al. 2024; An et al. 2025). Dysregulation of HIF‐1 is linked to metabolic diseases including MASLD (Huang et al. 2024; Yang et al. 2021). Accumulating evidence suggests that aberrant activation of the HIF‐1α/vascular endothelial growth factor (VEGF) signaling axis contributes to hepatic lipid accumulation, inflammation, pathological angiogenesis, and fibrosis during MASLD progression (Li et al. 2023). Importantly, hypoxia signaling not only acts as a downstream consequence of metabolic stress but also serves as an upstream driver that exacerbates metabolic imbalance and inflammatory responses in the liver (Fu et al. 2024).

Based on network pharmacology analysis, HIF‐1α/VEGF was identified as a core pathway mediating the protective effects of DHQ against MASLD (Filippovich et al. 2025). Distinct from previously reported AMPK‐ or inflammasome‐centered mechanisms, this finding highlights a novel regulatory axis linking hypoxia signaling, angiogenesis, and metabolic homeostasis. Accordingly, the present study combined network pharmacology with in vivo and in vitro experiments to validate the role of this pathway in DHQ‐mediated hepatoprotection.

## 2 Materials and Methods

### 2.1 Network Pharmacology Analysis

#### 2.1.1 Identification of Shared Targets Between MASLD and DHQ

MASLD‐related targets were sourced from GeneCards (https://www.genecards.org/), while the SMILES structure of DHQ was retrieved from PubChem (https://pubchem.ncbi.nlm.nih.gov/). Potential DHQ targets were predicted using Super‐PRED (https://prediction.charite.de/subpages/target_prediction.php) and TargetNet (http://targetnet.scbdd.com/calcnet/index/) platforms. Overlapping targets were identified using Venny 2.1 (https://bioinfogp.cnb.csic.es/tools/venny/).

#### 2.1.2 Protein–Protein Interaction (PPI) Network Analysis

Shared targets were uploaded to STRING (https://string‐db.org/) for PPI network data retrieval and visualized using Cytoscape 3.10.3. Key core targets were identified using the BottleNeck method.

#### 2.1.3 Gene Ontology (GO) and Kyoto Encyclopedia of Genes and Genomes (KEGG) Pathway Analysis

Shared targets were analyzed for GO and KEGG enrichment using the DAVID database (https://david.ncifcrf.gov/). The top 10 genes for GO annotation and top 20 pathways for KEGG enrichment were selected. Visualizations were created using the Bioinformatics Online platform (http://www.bioinformatics.com.cn).

### 2.2 Molecular Docking

The three‐dimensional (3D) structures of the core target proteins (AKT1, ESR1, PPARG, HIF‐1A, and MMP9) were obtained from the Protein Data Bank (PDB, https://www.rcsb.org/). The DHQ molecular structure in SDF format was downloaded from PubChem and converted to PDBQT format using OpenBabel. Protein structures were prepared by removing water molecules and adding polar hydrogens with AutoDockTools 1.5.6. Docking simulations were performed using AutoDock Vina 1.1.2 to evaluate the binding affinity and predict the optimal binding conformations between DHQ and target proteins. The docking grid boxes were defined based on the active or ligand‐binding sites of each protein. Binding poses with the lowest binding energy were selected for further analysis. PyMOL 2.5 was used to visualize and analyze molecular interactions. Binding energies were extracted and summarized in a heatmap to compare affinities across targets.

### 2.3 Animal Experiment

Male C57BL/6J mice (6–8 weeks, 18–22 g, 24 mice) were obtained from Beijing SPF Biotechnology Co. Ltd. Mice were housed in a controlled environment (22°C ± 2°C, 55% ± 5% humidity, 12‐h light/dark cycle).

After 1 week of adaptation to a regular diet, the mice were randomly assigned to one of four groups: Control group (Control), MASLD group (Model), DHQ low dose group (40 mg/kg) (DHQ‐L), and DHQ high dose group (80 mg/kg) (DHQ‐H), with 6 mice in each group. The dosages of DHQ were selected based on previous studies (Xu et al. 2023), which demonstrated effective biological activity and safety in mice. These doses were therefore chosen to evaluate the dose‐dependent effects of DHQ in this study. The Control group received a regular diet, while the other three groups were provided with a high‐fat diet for 4 weeks to generate the MASLD model (Lee et al. 2010). After the 4‐week pre‐feeding period, the mice were treated by intraperitoneal injection (0.01 mL/g) once daily for 6 consecutive weeks. The HFD was continuously maintained throughout the subsequent 6‐week intervention period until the end of the experiment. The Control group was injected daily with physiological saline (ST341, Beyotime, Shanghai, China), while the MASLD Model group was injected with corn oil. The low and high dose DHQ groups were injected daily with their respective doses of DHQ in a corn oil solution. During the experiment, the mice were observed daily, and their body weight was measured on a weekly basis. The daily food consumption of the mice was quantified and documented at 48‐h intervals. Following the intervention, the animals were subjected to a 24‐h fast without access to water. Blood samples were collected via orbital sinus puncture, centrifuged at 3000 rpm for 10 min to isolate serum. Mice were then humanely sacrificed by cervical dislocation, and their livers were harvested. The liver was washed with saline, blotted dry, photographed, and weighed. A portion was preserved in 4% paraformaldehyde, and the residual part was placed in Eppendorf (EP) tubes for further experiments.

### 2.4 Cell Culture and Treatment

The human hepatocellular carcinoma cell line 2 (HepG2) (CL‐0103) was purchased from Wuhan Puno Sai Life Technology Co. Ltd. The cells were maintained in Dulbecco's Modified Eagle Medium (DMEM) (11995, Solarbio, Beijing, China), enriched with 10% fetal bovine serum (C0226, Beyotime, Shanghai, China) and 1% penicillin–streptomycin (PB180120, Pricella, Wuhan, China), at 37°C with 5% CO_2_. Cells undergoing logarithmic proliferation were chosen to be used in the experimental assays. HepG2 cells were STR‐authenticated and tested for mycoplasma contamination, and the results showed no contamination. Although HepG2 cells are derived from hepatocellular carcinoma, they are widely used to model hepatic lipid accumulation and inflammatory responses in vitro.

The hypoxia‐inducible factor 1 (HIF‐1) agonist cobalt chloride (CoCl_2_) (232696‐5G, Sigma‐Aldrich, St. Louis, MO, USA) and oleic acid (Purity 99%, ST2053, Solarbio, Beijing, China) were used in the experiments. The in vitro MASLD model was established by treating HepG2 cells with 0.5 mmol/L oleic acid, as previously described (Wang, Sheng, et al. 2021). Six experimental groups were set: Control group (Control), MASLD Model group (Model), DHQ low dose group (DHQ‐L), DHQ high dose group (DHQ‐H), CoCl_2_ agonist group (CoCl_2_), and DHQ high dose + CoCl_2_ agonist group (DHQ‐H + CoCl_2_), with at least three replicates in each group. The low and high‐dose DHQ groups received 0.5 mmol/L oleic acid treatment, with the addition of DHQ at final concentrations of 50 and 100 μmol/L, respectively, based on the concentration of the stock solution (prepared in dimethyl sulfoxide) (Xu et al. 2023). The CoCl_2_ group was treated with DMEM containing 100 μM CoCl_2_ (as a HIF‐1 agonist) (Chen et al. 2020), and the DHQ‐H + CoCl_2_ group received the same concentration of CoCl_2_ as the CoCl_2_ group, in addition to high‐dose DHQ. Following 24 h of treatment, the cells were harvested for subsequent experiments.

### 2.5 Histological Analysis

Liver tissues, after fixation, were subjected to conventional paraffin embedding, sectioned, and stained with Hematoxylin and Eosin (H&E) dye (C0105S, Beyotime, Shanghai, China) as per established protocols. Histological changes were then examined under an inverted fluorescence microscope (CKX53, OLYMPUS, Tokyo, Japan).

### 2.6 Biochemical Index Measurement

In strict adherence to the manufacturer's instructions, serum concentrations of total cholesterol (TC), low‐density lipoprotein cholesterol (LDL‐C), and high‐density lipoprotein cholesterol (HDL‐C), alanine aminotransferase (ALT), aspartate aminotransferase (AST), and triglycerides (TG) in mice were measured (A111‐1‐1, A113‐1‐1, A112‐1‐1, C009‐2‐1; C010‐2‐1; A110‐1‐1, Nanjing Jiancheng, Nanjing, China). Furthermore, the TG concentrations in the cells of each experimental group were also evaluated.

### 2.7 Enzyme‐Linked Immunosorbent Assay (ELISA)

The concentrations of the tumor necrosis factor‐α (TNF‐α), interleukin‐6 (IL‐6), inflammatory cytokines interleukin‐1β (IL‐1β) and IL‐18 in mouse serum (CB10851; CB10187; CB10173; CB10172, COIBO BIO, Shanghai, China) and in cell supernatants (CB11762; CB10373; CB10347; CB10313, COIBO BIO, Shanghai, China) were measured using the corresponding ELISA kits. Absorbance was recorded at 450 nm with a microplate reader (CMaxPlus, MOLECULAR DEVICES, USA). A standard curve was generated with standard concentrations plotted against optical density (OD) values, and the cytokine levels in both the serum and supernatants were determined based on the OD values of the samples.

### 2.8 Cellular Lipid Accumulation Observation

HepG2 cells (1 × 10^5^ cells) were first immobilized with 4% paraformaldehyde for 30 min, washed twice with PBS, and lipid accumulation was assessed using Oil Red O staining (C0157S, Beyotime, Shanghai, China) according to the manufacturer's instructions. After staining, cells were mounted with glycerol gelatin and visualized for lipid deposits under an inverted microscope (XD‐202, by Jiangnan, Nanjing, China).

### 2.9 Real‐Time Quantitative Polymerase Chain Reaction (RT‐qPCR)

Total RNA from HepG2 cells or liver tissue was extracted using TRIzol reagent (R0016, Beyotime, Shanghai, China). cDNA synthesis was carried out with the mRNA First Strand cDNA Synthesis Kit (D7178M, Beyotime, Shanghai, China). PCR amplification was conducted using the SYBR Green One‐Step qRT‐PCR Kit (D7268S, Beyotime, Shanghai, China) on a fluorescence quantitative PCR instrument (LightCycler96, Roche, Switzerland). Glyceraldehyde‐3‐phosphate dehydrogenase (GAPDH) was used as the reference gene. The data were analyzed using the 2^−ΔΔ*CT*^ method. Primer sequences are provided in Table 1.

**TABLE 1:** Primer sequences for RT‐qPCR.

Gene name	Species	Sequence (5′–3′)
*TNF‐α*	Human	F: CTCTTCTGCCTGCTGCACTTTG R: ATGGGCTACAGGCTTGTCACTC
	Mouse	F: GGTGCCTATGTCTCAGCCTCTT R: GCCATAGAACTGATGAGAGGGAG
*IL‐6*	Human	F: AGACAGCCACTCACCTCTTCAG R: TTCTGCCAGTGCCTCTTTGCTG
	Mouse	F: TACCACTTCACAAGTCGGAGGC R: CTGCAAGTGCATCATCGTTGTTC
*IL‐1β*	Human	F: CCAGGGACAGGATATGGAGCA R: TTCAACACGCAGGACAGGTACAG
	Mouse	F: ATGATGGCTTATTACAGTGGCAA R: GTCGGAGTTCGTAGCTGGA
*IL‐18*	Human	F: TCTACTGGTTCAGCAGCAGCCATCTTTA R: CTGCCACCTGCTGCAGTCTA
	Mouse	F: GACTCTTGCGTCAACTTCAAGG R: CAGGCTGTCTTTTGTCAACGA
*HIF‐1α*	Human	F: TGCAACATGGAAGGTATTGC R: TTCACAAATCAGCACCAAGC
	Mouse	F: CCTGCACTGAATCAAGAGGTTGC R: CCATCAGAAGGACTTGCTGGCT
*VEGF*	Human	F: GGCAGAATCATCACGAAGT R: CACAGGATGGCTTGAAGAT
	Mouse	F: TCACCAAAGCCAGCACATAG R: AATGCTTTCTCCGCTCTGAA
*LKB1*	Human	F: CTACTGAGGAGGTTACGGCACA R: ACGCTGTCCAGCATTTCCTGCA
	Mouse	F: GCCTGGAATACCTACACAGCCA R: GCAGGTGTCATCCACAGCGAAA
*AMPKα1*	Human	F: AGGAAGAATCCTGTGACAAGCAC R: CCGATCTCTGTGGAGTAGCAGT
	Mouse	F: GGTGTACGGAAGGCAAAATGGC R: CAGGATTCTTCCTTCGTACACGC
*ACC*	Human	F: TTCACTCCACCTTGTCAGCGGA R: GTCAGAGAAGCAGCCCATCACT
	Mouse	F: GTTCTGTTGGACAACGCCTTCAC R: GGAGTCACAGAAGCAGCCCATT
*GAPDH*	Human	F: TTCTCAGCCTTGACGGTGC R: AAGGTCGGAGTCAACGGATTT
	Mouse	F: TGATTCTACCCACGGCAAGT R: AGCATCACCCCATTTGATGT

### 2.10 Western Blot (WB)

Liver tissues or cells were lysed with radioimmunoprecipitation assay (RIPA) lysis buffer (P0013B, Beyotime, Shanghai, China) to extract total protein. Protein concentration was measured using the bicinchoninic acid (BCA) Protein Assay Kit (P0009, Beyotime, Shanghai, China). Protein samples were separated by 10% sodium dodecyl sulfate‐polyacrylamide gel electrophoresis (SDS‐PAGE; P0015A, Beyotime, Shanghai, China) and transferred to polyvinylidene fluoride (PVDF) membrane (P0021S, Beyotime, Shanghai, China). After blocking with 5% non‐fat milk for 1.5 h, the membrane was incubated overnight with primary antibodies: Liver Kinase B1 (LKB1) (ab199970), Phosphorylated Liver Kinase B1 (p‐LKB1) (ab63473), AMP‐activated Protein Kinase Alpha Subunit (AMPK‐α) (ab32047), Phosphorylated AMPK‐α (p‐AMPK‐α) (ab133448), Acetyl‐CoA Carboxylase (ACC) (ab45174), p‐ACC (ab68191) (all primary antibodies diluted at 1:1000, Abcam, Cambridge, MA, USA), HIF‐1α (1:1000, GB111339‐100, Servicebio, Wuhan, China), vascular endothelial growth factor (VEGF) (1:1000, GB114200‐100, Servicebio, Wuhan, China), and GAPDH (1:1000, GB15002‐100, Servicebio, Wuhan, China). The membrane was incubated with secondary antibody (1:2000 dilution, ZB‐2305, ZSGB‐BIO, Beijing, China) for 2 h. Protein bands were detected using the enhanced chemiluminescence (ECL) kit (35055, Pierce, Thermo Fisher, Waltham, MA, USA). GAPDH served as the loading control and signal intensity was analyzed with ImageJ (Ver.1.52b, NIH, Bethesda, MD, USA).

### 2.11 Statistical Analysis

Data were analyzed with GraphPad Prism software (version 9.5, GraphPad Software, La Jolla, CA, USA). The *t*‐test was used for comparisons between two groups, while one‐way analysis of variance (ANOVA) was applied for multiple group comparisons. Post hoc analysis was conducted using the Tukey method. Results are expressed as mean ± standard deviation, with *p* < 0.05 indicating statistical significance.

## 3 Results

### 3.1 Identification of Common Targets Between DHQ and MASLD

**FIGURE 1:**
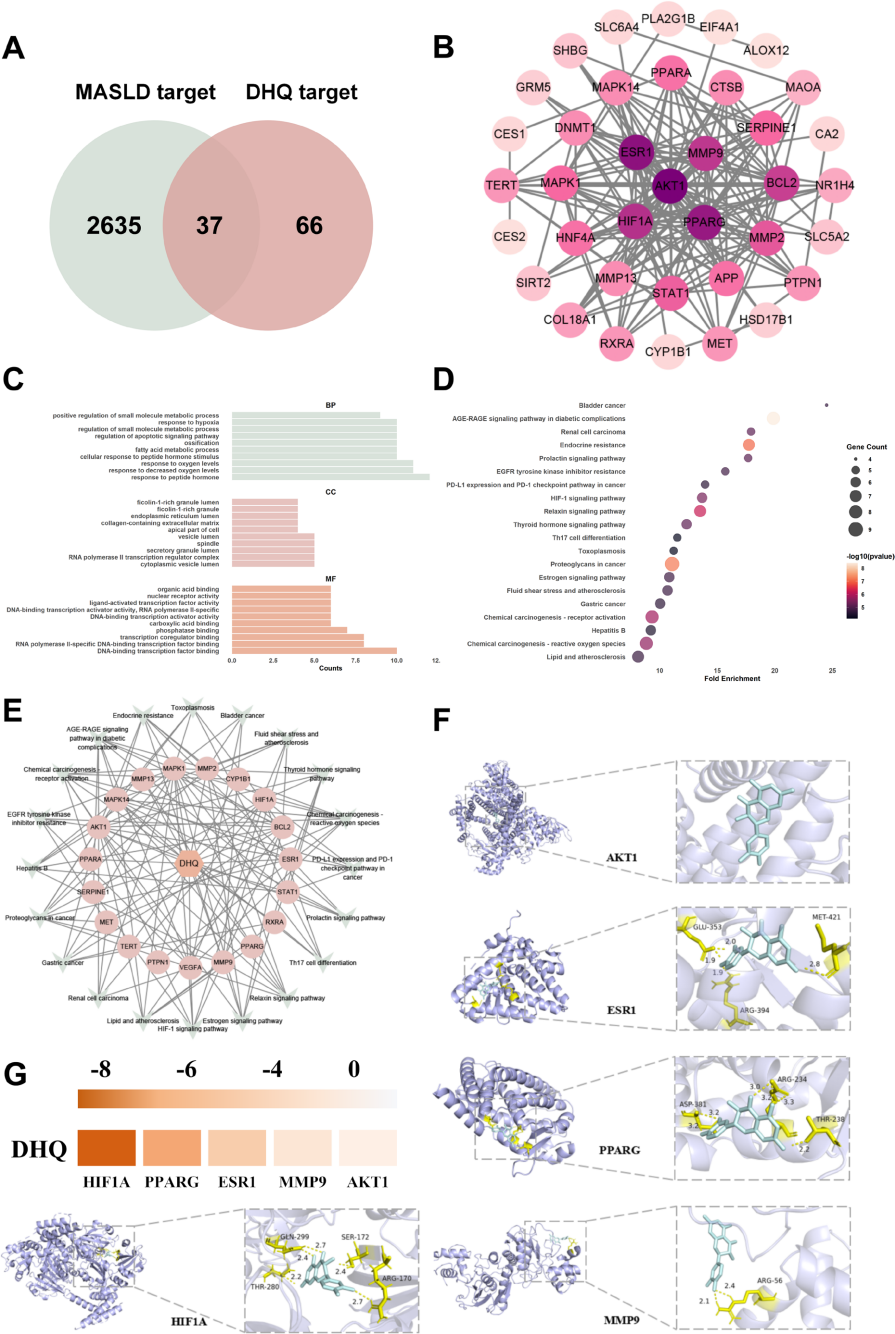
Integrated analysis based on network pharmacology and molecular docking. (A) Identification of the overlapping targets between DHQ and disease‐related genes. (B) PPI network of the common targets. (C) GO enrichment analysis. (D) KEGG pathway enrichment analysis of the common targets. (E) Drug‐target‐pathway interaction network diagram illustrating the regulatory relationship among DHQ, its predicted targets, and key signaling pathways. (F) Molecular docking models showing the binding interactions between DHQ and core target proteins. (G) Binding energy heatmap of DHQ with key target proteins.

By querying the GeneCards database, a set of 2635 targets associated with MASLD was retrieved, and through searches in the Super‐PRED and TargetNet databases, 66 targets linked to DHQ were pinpointed; subsequently, an intersection analysis was conducted, revealing 37 shared targets between MASLD and DHQ (Figure 1A). Utilizing these shared targets, a PPI network was constructed to elucidate the intermolecular relationships (Figure 1B). The top five genes with the highest scores were identified using the BottleNeck method, which included RAC‐alpha serine/threonine‐protein kinase 1 (AKT1), estrogen receptor 1 (ESR1), peroxisome proliferator‐activated receptor gamma (PPARG), HIF‐1A, and matrix metalloproteinase 9 (MMP9).

### 3.2 GO and KEGG Analysis

The 37 common targets of DHQ and MASLD were analyzed for biological processes (BP), cellular components (CC), and molecular functions (MF). The top 10 common targets and top 20 pathways (Figure 1C,D) were listed. Regarding biological processes, these targets were primarily involved in responses to peptide hormones, changes in oxygen levels, and oxygen level fluctuations. In terms of cellular components, the common targets were linked to the cytoplasmic vesicle lumen and others, and for molecular functions, they were linked to activities including transcription coregulator binding, nuclear receptor activity, and ligand‐activated transcription factor activity. Additionally, KEGG pathway analysis uncovered the signaling pathways linked to these targets, with the top 20 pathways presented in Figure 1D. The drug‐target‐signaling pathway interaction analysis was constructed and visualized using Cytoscape 3.9.1, with the results shown in Figure 1E. Among these, the HIF‐1 signaling pathway was chosen for further validation.

To further validate these core targets, molecular docking was performed between DHQ and the selected hub proteins, and representative docking poses are illustrated in Figure 1F. The binding energy data summarized in the heatmap (Figure 1G) demonstrate strong and stable interactions, particularly with HIF‐1A, PPARG, and ESR1, indicating their potential as primary targets of DHQ.

### 3.3 Effects of DHQ on MASLD Mice

**FIGURE 2:**
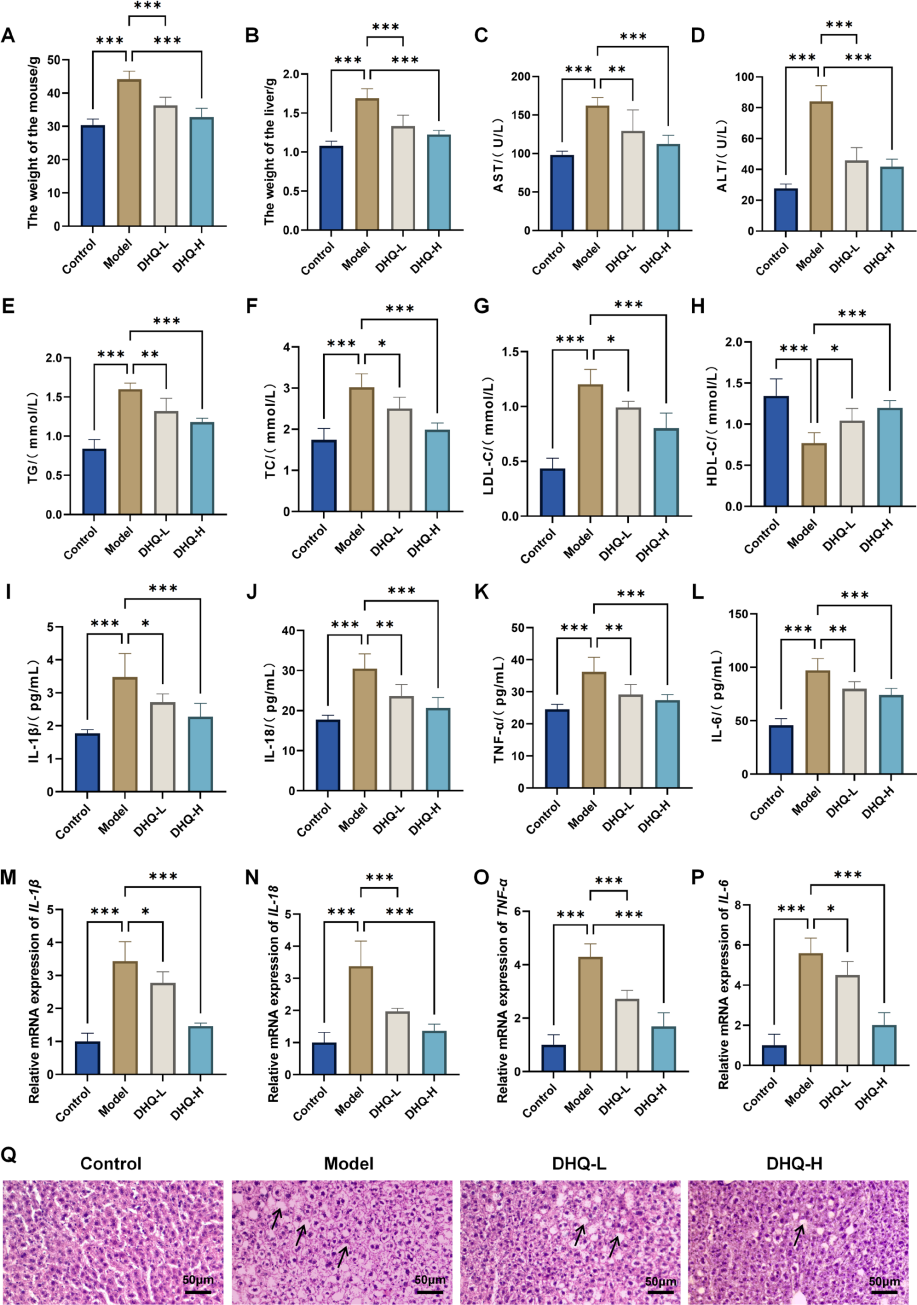
Effects of DHQ on MASLD mice. (A, B) Body weight and liver weight of mice in each group. (C–H) Serum levels of AST, ALT, TG, TC, LDL‐C, and HDL‐C. (I–L) Serum concentrations of inflammatory cytokines IL‐1β, IL‐18, TNF‐α, and IL‐6 measured by ELISA. (M–P) Relative mRNA expression levels of inflammatory cytokines IL‐1β, IL‐18, TNF‐α, and IL‐6 in mouse liver tissues. (Q) Representative H&E staining images of liver tissue sections. Data are expressed as mean ± SD, *n* = 6; *p < 0.05, **p < 0.01, ***p < 0.001.

The impact of DHQ on body weight, liver weight, serum biochemical parameters, inflammatory cytokines, and liver histopathology was comprehensively assessed in MASLD mice. Compared with the Control group, mice in the Model group exhibited significantly increased body and liver weights (*p* < 0.001; Figure 2A,B). Serum biochemical analysis revealed marked elevations in AST, ALT, TG, TC, and LDL‐C levels, along with a significant reduction in HDL‐C (*p* < 0.001; Figure 2C–H), indicating hepatic injury and lipid metabolic imbalance. DHQ treatment significantly decreased both body and liver weights (*p* < 0.001), reduced serum AST, ALT, TG, TC, and LDL‐C levels, and restored HDL‐C levels (*p* < 0.05), suggesting a protective effect against MASLD‐related metabolic disturbances.

Additionally, the Model group showed pronounced increases in serum IL‐1β, IL‐18, TNF‐α, and IL‐6 concentrations (*p* < 0.05; Figure 2I–L), as well as significantly elevated hepatic mRNA expression levels of these cytokines (*p* < 0.05; Figure 2M–P), indicating enhanced systemic and local inflammatory responses. DHQ administration led to a significant suppression of both serum cytokine levels and hepatic mRNA expression, demonstrating that DHQ effectively attenuates inflammation at both the transcriptional and protein levels.

H&E staining further confirmed that DHQ treatment alleviated hepatic steatosis and structural disorganization, as evidenced by reduced lipid vacuole formation (as indicated by the arrows) and improved hepatocellular architecture (Figure 2Q).

These findings collectively indicate that DHQ mitigates MASLD progression by regulating lipid metabolism, reducing inflammation, and preserving liver histology.

### 3.4 DHQ Modulates Lipid Metabolism‐Related Proteins and Inhibits HIF‐1α/VEGF Signaling Pathway in MASLD Mice

**FIGURE 3:**
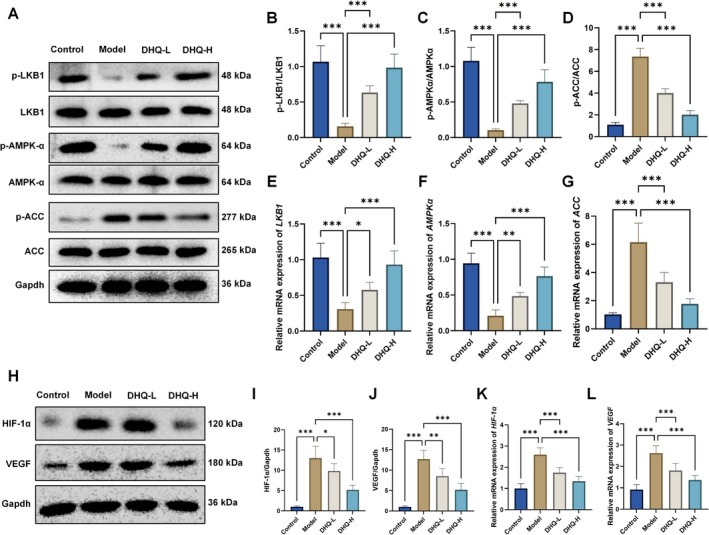
Effects of DHQ on the expression of lipid metabolism‐related proteins and the HIF‐1α/VEGF signaling pathway in the liver tissue of MASLD mice. (A–G) Protein and mRNA expression levels of ACC, AMPK, and LKB1. (H–L) Protein and mRNA expression levels of HIF‐1α and VEGF. Data are expressed as mean ± SD, *n* = 6; *p < 0.05, **p < 0.01, ***p < 0.001.

Western blot and RT‐qPCR analyses showed that DHQ treatment significantly regulated key proteins and genes involved in lipid metabolism in the liver tissue of MASLD mice. Compared to the Control group, the Model group exhibited a marked increase in ACC protein expression and significant decreases in AMPK and LKB1 expression (*p* < 0.001; Figure 3A–G). DHQ administration at both low and high doses effectively reversed these alterations by downregulating ACC and upregulating AMPK and LKB1 protein and mRNA levels (*p* < 0.05), indicating improvement in hepatic lipid metabolism.

Additionally, the expression of HIF‐1α and VEGF, critical components of the HIF‐1 signaling pathway, was significantly upregulated in MASLD model mice compared to controls (*p* < 0.001; Figure 3H–L), suggesting pathway activation during disease progression. Treatment with DHQ significantly suppressed HIF‐1α and VEGF protein and mRNA expression levels (*p* < 0.05), indicating that DHQ may alleviate MASLD in part through blockade of the HIF‐1α/VEGF pathway.

### 3.5 DHQ Improves Lipid Metabolism and Inhibits Inflammation in HepG2 Cells, With CoCl_2_ Reversing Its Effects

**FIGURE 4:**
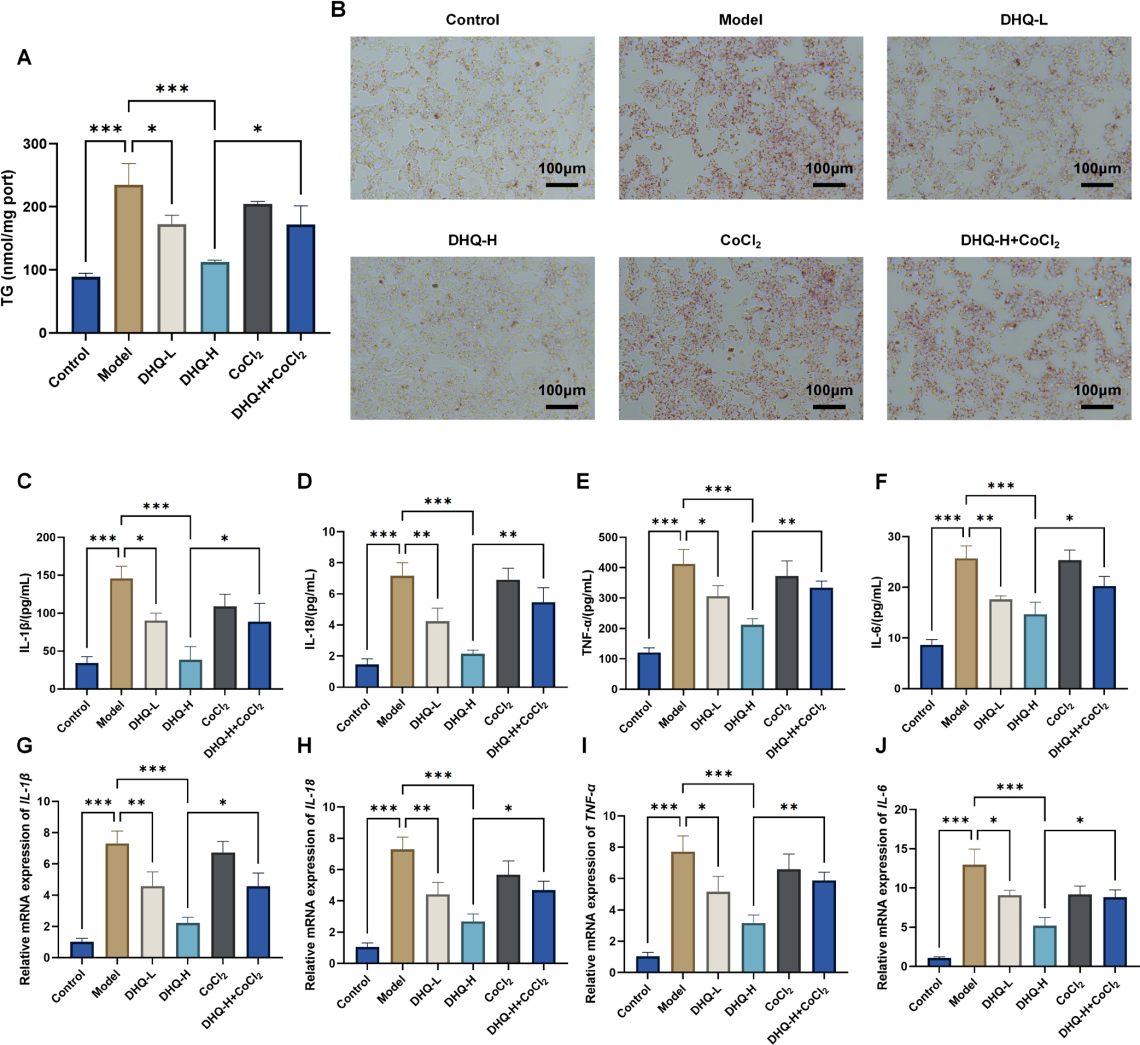
Effects of DHQ on lipid accumulation and inflammatory responses in HepG2 cells. (A) TG content. (B) Oil Red O staining. (C–F) ELISA measurement of IL‐1β, IL‐18, TNF‐α, and IL‐6 protein levels. (G–J) RT‐qPCR analysis of IL‐1β, IL‐18, TNF‐α, and IL‐6 mRNA expression. Data are shown as mean ± SD, *n* = 3; *p < 0.05, **p < 0.01, ***p < 0.001.

TG content and Oil Red O staining showed that the Model group had significantly increased lipid accumulation compared to Control (*p* < 0.001; Figure 4A,B). DHQ treatment significantly reduced TG levels and lipid droplets (*p* < 0.05), while co‐treatment with CoCl_2_, a HIF‐1α stabilizer, partially reversed these effects (*p* < 0.05). ELISA results indicated elevated IL‐1β, IL‐18, TNF‐α, and IL‐6 in the Model group (*p* < 0.001; Figure 4C–F), which were significantly decreased by DHQ treatment (*p* < 0.001). RT‐qPCR confirmed similar trends in mRNA expression of these cytokines (*p* < 0.05; Figure 4G–J). CoCl_2_ co‐treatment partly abrogated the anti‐inflammatory effects of DHQ. These data suggest that DHQ improves lipid metabolism and suppresses inflammation in HepG2 cells, potentially via modulation of the HIF‐1α pathway.

### 3.6 DHQ Regulates Lipid Metabolism and HIF‐1α/VEGF Signaling Pathway in HepG2 Cells

**FIGURE 5:**
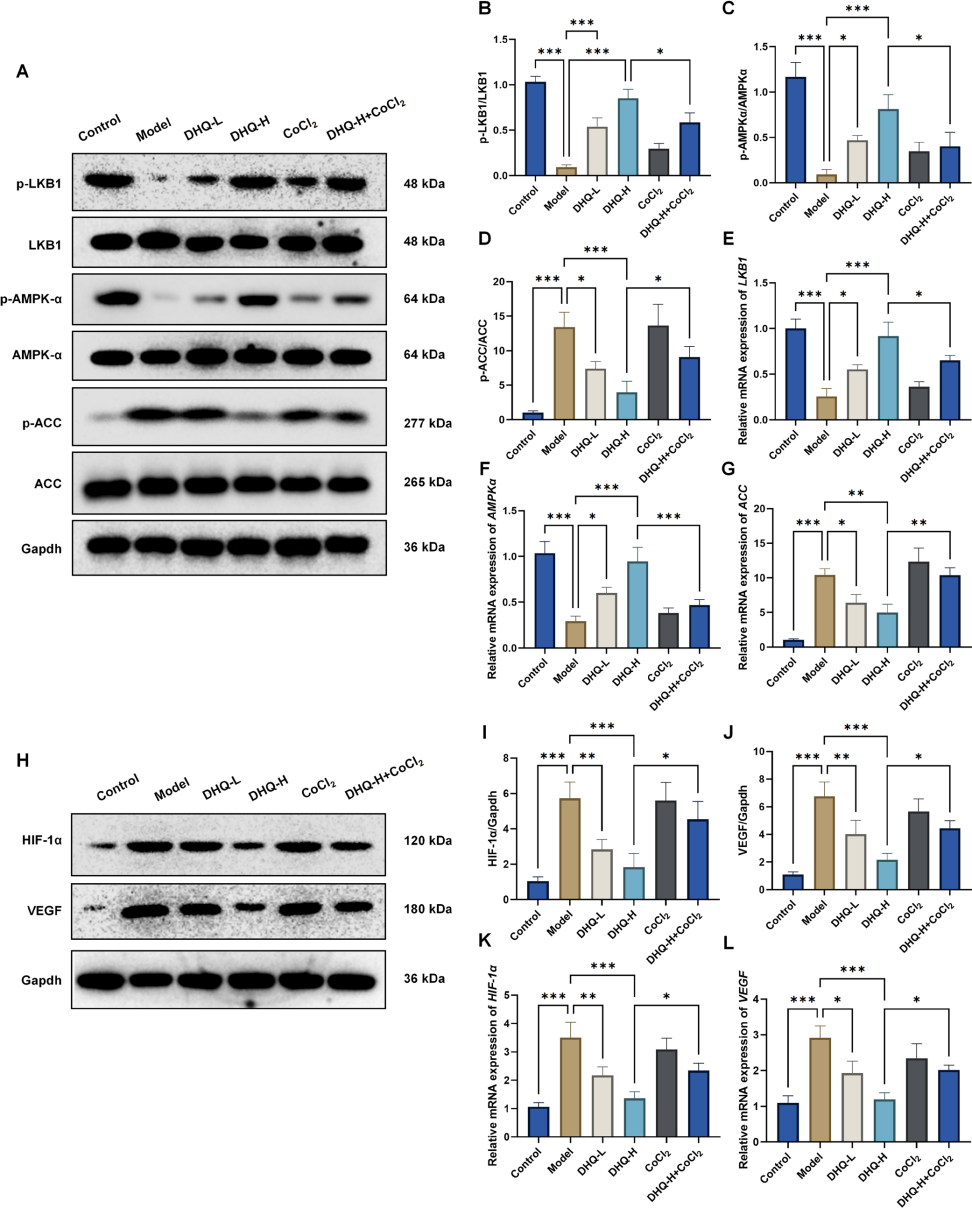
Effects of DHQ on the expression of lipid metabolism‐related proteins and the HIF‐1α/VEGF signaling pathway in HepG2 cells. (A–G) Protein and mRNA expression levels of p‐LKB1, p‐AMPK, and p‐ACC. (H–L) Protein and mRNA expression levels of HIF‐1α and VEGF. Data are expressed as mean ± SD, *n* = 3; *p < 0.05, **p < 0.01, ***p < 0.001.

Western blot and RT‐qPCR analyses demonstrated that, compared to the Control group, MASLD model cells showed significantly decreased expression of p‐LKB1 and p‐AMPK and increased expression of p‐ACC (*p* < 0.001; Figure 5A–G). Treatment with DHQ‐L and DHQ‐H significantly reversed these changes by upregulating p‐LKB1 and p‐AMPK and downregulating p‐ACC (*p* < 0.05). CoCl_2_ co‐treatment inhibited the DHQ‐induced modulation of these proteins, indicating that CoCl_2_ attenuates the inhibitory effect of DHQ on lipid synthesis. At the mRNA level, consistent trends were observed (Figure 5E–G).

Regarding the HIF‐1α/VEGF pathway, the Model group exhibited significant upregulation of HIF‐1α and VEGF protein and mRNA expression compared to Control (*p* < 0.001; Figure 5H–L). DHQ treatment significantly downregulated HIF‐1α and VEGF expression (*p* < 0.05), whereas CoCl_2_ co‐treatment partially reversed this suppression. These results suggest that DHQ may alleviate MASLD progression by modulating lipid metabolism and inhibiting activation of the HIF‐1α/VEGF signaling pathway.

## 4 Discussion

This study employed network pharmacology to identify the potential targets and pathways through which DHQ may exert therapeutic effects on MASLD. Thirty‐seven common targets were identified, among which five core targets—AKT1, ESR1, PPARα, HIF‐1A, and MMP9—were linked to the regulation of lipid metabolism, insulin resistance, oxidative stress, and inflammatory processes. KEGG analysis revealed that the HIF‐1 signaling pathway was one of the greatest significance pathways involved, suggesting its pivotal role in DHQ's therapeutic effects on MASLD. As a pivotal transcription factor, HIF‐1 participates in the cellular adaptive reactions to low‐oxygen environments, modulates metabolic processes, and assumes a vital function in the pathogenesis and progression of liver and metabolic disorders (Cui et al. 2023). In MASLD, excessive activation of HIF‐1α is closely related to hepatic lipid deposition and fibrosis development (Mesarwi et al. 2021; Qiu et al. 2017). Accordingly, to gain a more in‐depth and definitive understanding of the therapeutic impacts of DHQ in treating MASLD, we focused on the HIF‐1 signaling pathway and conducted experiments to investigate whether DHQ alleviates MASLD‐related pathological changes by modulating this pathway.

ALT and AST are key enzymes involved in liver metabolism, and their elevated serum levels serve as reliable indicators of hepatocellular injury (Aubrecht et al. 2025). TG, primarily synthesized in the liver, reflects lipid metabolic status (Wang, Sheng, et al. 2021). In this study, MASLD mice exhibited significant increases in ALT, AST, and TG levels, accompanied by severe hepatic steatosis and lipid accumulation, confirming successful model induction. DHQ treatment notably reduced serum ALT, AST, and TG levels and alleviated hepatic histopathological damage, suggesting that DHQ may improve hepatocellular integrity and lipid metabolic homeostasis, both of which are central to MASLD pathogenesis. Both IL‐1β and IL‐18 are crucial in triggering the release of various inflammatory mediators, which in turn initiate pathological inflammation (Kodi et al. 2024). In the present study, serum concentrations of IL‐1β, IL‐18, TNF‐α, and IL‐6 were significantly elevated in MASLD mice, indicating an enhanced inflammatory response. DHQ treatment markedly reduced the levels of these cytokines; given that chronic inflammation plays a critical role in driving MASLD progression, the observed reduction in IL‐1β, IL‐18, TNF‐α, and IL‐6 levels further indicates that DHQ may mitigate inflammation‐associated liver injury, thereby exerting a dual regulatory effect on metabolic and inflammatory pathways relevant to MASLD.

AMPK is a key regulator of energy homeostasis and lipid metabolism, activated by its upstream kinase LKB1 under energy stress to maintain hepatic lipid balance by promoting fatty acid oxidation and inhibiting lipid synthesis (Huang et al. 2021; Lan et al. 2021). In MASLD mouse livers, AMPK and LKB1 expression were decreased, while ACC, involved in lipid synthesis, was increased. DHQ treatment reversed these changes, suggesting that DHQ may restore hepatic energy sensing and lipid metabolic homeostasis through activation of the AMPK signaling axis, a pathway critically involved in MASLD pathogenesis. Additionally, HIF‐1α, implicated in MASLD progression through promoting oxidative stress, inflammation, and lipid accumulation, along with its downstream effector VEGF, which aggravates hepatic pathology, were both downregulated by DHQ (Wang, Liu, et al. 2021; Luo et al. 2023). Given the established role of the HIF‐1α/VEGF pathway in linking metabolic stress to inflammatory and fibrotic responses in MASLD, these findings indicate that DHQ may exert hepatoprotective effects by modulating hypoxia‐related pathological signaling. Similar regulatory effects of DHQ on this pathway were observed in HepG2 cells, supporting its therapeutic potential in MASLD, further supporting the mechanistic relevance and translational potential of DHQ in MASLD intervention.

In vitro HepG2 experiments using the HIF‐1 activator CoCl_2_ demonstrated that CoCl_2_ attenuated DHQ‐induced downregulation of lipid synthesis‐related factors (e.g., ACC) and inflammatory cytokines (e.g., IL‐1β), indicating that activation of HIF‐1α signaling can counteract the metabolic and anti‐inflammatory effects of DHQ. Additionally, CoCl_2_ partially reversed DHQ's inhibition of inflammasome components, including IL‐1β, and IL‐18, further supporting the involvement of HIF‐1α‐dependent inflammatory signaling in DHQ‐mediated regulation. These findings suggest that the HIF‐1α/VEGF pathway plays a critical role in mediating DHQ's therapeutic effects on lipid metabolism and inflammation in MASLD, highlighting DHQ as a promising candidate for MASLD treatment, providing mechanistic evidence that DHQ may exert its protective effects at least in part by targeting hypoxia‐associated inflammatory and metabolic pathways, thereby reinforcing its potential as a multi‐target therapeutic candidate for MASLD.

## 5 Conclusion

This study reveals that DHQ ameliorates MASLD by regulating lipid metabolism and inflammation via AMPK and HIF‐1α/VEGF pathways. DHQ activates AMPK and LKB1, inhibits ACC, and suppresses IL‐1β and IL‐18 secretion, reducing liver inflammation. It also mitigates MASLD pathology by inhibiting HIF‐1α activation. DHQ may represent a promising therapeutic candidate for MASLD, warranting further clinical investigation. However, the present findings are mainly derived from experimental animal and cell models, and further clinical validation is required to confirm the therapeutic efficacy and translational potential of DHQ in human MASLD. In addition, the long‐term safety and optimal dosing strategy of DHQ warrant further investigation. Overall, DHQ shows promise as a therapeutic agent for MASLD and merits further exploration in future translational and clinical studies.

## Author Contributions

**Xinyue Zhang:** conceptualization (equal), investigation (equal), methodology (equal), data curation (equal), formal analysis (equal), writing – original draft (equal), writing – review and editing (equal). **Han Zhang:** conceptualization (equal), investigation (equal), methodology (equal), data curation (equal), formal analysis (equal), writing – original draft (equal), writing – review and editing (equal). **Bing Wu:** conceptualization (equal), investigation (equal), methodology (equal), data curation (equal), formal analysis (equal), writing – original draft (equal), writing – review and editing (equal). **Pengfei Wu:** investigation (equal), methodology (equal), data curation (equal), formal analysis (equal), validation (equal), writing – original draft (equal), writing – review and editing (equal). **Ao Shen:** investigation (equal), methodology (equal), data curation (equal), resources (equal), writing – review and editing (equal). **Cheng Zhang:** conceptualization (equal), supervision (equal), resources (equal), project administration (equal), writing – review and editing (equal). **Yuqiao Zeng:** data curation (equal), formal analysis (equal), resources (equal), writing – review and editing (equal). **Yanjun Wang:** investigation (equal), methodology (equal), resources (equal), writing – review and editing (equal). **Hao Xu:** conceptualization (equal), supervision (equal), resources (equal), project administration (equal), writing – review and editing (equal). **Yiyu He:** conceptualization (equal), supervision (equal), resources (equal), project administration (equal), writing – review and editing (equal). **Likun Wang:** conceptualization (equal), supervision (equal), resources (equal), project administration (equal), writing – review and editing (equal).

## Funding

This research was funded by the Natural Science Foundation of Shandong Province (ZR2021MH375), TCM Science and Technology Key Project of Shandong Province (Z‐2022036), and Key Research and Development Program of Linyi City (2025YX0031).

## Ethics Statement

All animal procedures were approved by the Science and Technology Ethics Committee, Linyi People's Hospital, with the approval number 202411‐A‐008.

## Conflicts of Interest

The authors declare no conflicts of interest.

## Data Availability

The data that support the findings of this study are available from the corresponding author upon reasonable request.
